# Challenges and limitations for live cell imaging in extreme cold

**DOI:** 10.1088/2050-6120/ae8c12

**Published:** 2026-07-29

**Authors:** Anne-Pia M Marty, Amir Rahmani, Francesca W van Tartwijk, Lloyd S Peck, Melody S Clark, Clemens F Kaminski

**Affiliations:** 1 Department of Chemical Engineering and Biotechnology, University of Cambridge, Cambridge CB3 0AS, United Kingdom; 2 British Antarctic survey High Cross, Madingley Road, Cambridge CB3 0ET, United Kingdom

**Keywords:** optical microscopy, super resolution, extremophiles

## Abstract

Many ecosystems thrive in near-0 °C conditions, and the mechanisms supporting life in these conditions remain understudied due to the challenges in reproducing such environments in laboratory conditions. One such example is polar organisms, that have adapted their entire lifecycle to operate below freezing temperatures through largely unknown cellular adaptations. As rapid polar warming threatens these species, elucidating their survival strategies is increasingly urgent. Fluorescence-based optical microscopy has been central to the understanding of the dynamic processes sustaining life at the cellular level, yet most imaging approaches have been developed and validated for conditions near mammalian physiological temperatures. Imaging at low temperature introduces a distinct physical regime in which molecular motion, membrane organisation, protein conformational dynamics, and fluorophore photophysics are fundamentally altered. As a result, imaging tools, fluorescent probes, and super-resolution methods optimised at 37 °C often fail when applied near 0 °C, or they report biased information. Here, we examine the conceptual, technical, and practical challenges associated with live-cell fluorescence microscopy at cold temperatures. We discuss when and why common imaging modalities and labelling strategies break down, and how probe behaviour becomes tightly coupled to local changes in physicochemical environment. We offer a perspective on new biological questions that become accessible for study with a microscopy platform optimised for imaging in cold conditions. We highlight trade-offs in current temperature-control strategies and identify unmet needs in fluorophore design, instrument engineering, and quantitative standards. By framing cold microscopy as a distinct operational regime rather than an extension of conventional live-cell imaging, this perspective aims to guide the development of robust tools for studying biological systems near-0 °C conditions.

## 1. Overview

Temperature influences a wide range of biological processes across multiple scales, including correct protein folding (Feller 2018), organelle integrity (Li *et al* 2018), cell stiffness and mobility (Wu *et al* 2014), and cancer tissue mechanics (Li *et al* 2015). Implementing sub-ambient temperature control during live-cell imaging would provide valuable insights into these phenomena. However, imaging live samples at sub-ambient temperatures poses different challenges to conventional imaging at 20 °C or 37 °C.

Most of the literature on imaging below ambient temperatures comes from static cryogenic (below −150 °C) imaging of vitrified samples (Wolff *et al* 2016). However, the challenges and opportunities associated with real-time live-cell imaging in the 0 °C–10 °C regime typical for polar environments cannot be inferred by extrapolation between room-temperature live imaging and cryogenic approaches (Yuan and Orrit 2014, Choi *et al* 2022). Antarctic marine species are highly sensitive to even 1 °C of ocean warming, yet paradoxically carry a heavy energetic burden due to cold denaturation effects on their proteostasis pathways (Peck 2016, Clark *et al* 2019). Thus, studying the dynamics of how proteins, cytoplasm and organelles perform at very cold temperatures is critical to our understanding of organism functioning, and survival, in extreme cold.

In biological systems adapted to the cold, molecular kinetics, transport processes, and material properties are altered (Yu and Barbara 2004, Dix and Verkman 2008). This, in turn, has repercussions on the behaviour and therefore choice of fluorescent probes, instrumentation, and experimental design.

Many systems have been developed to enable temperature-controlled microscopy. However, as optical setups are highly sensitive to perturbations, these solutions inevitably involve a trade-off between resolution and temperature range. Imaging at cold temperatures introduces specific challenges, including operating below the atmospheric dew point, which leads to condensation on optical surfaces. Additional challenges arise from changes in physical and optical properties of materials with temperature. Finally, constraints linked to the properties of photo-labels, including their stability at low temperature, introduce further considerations for fluorescent probe design.

These factors affect the feasibility of different microscopy techniques to be applied at low temperatures. In this perspective, we examine individual challenges in turn, and describe which microscopy modalities, including super-resolution imaging, can be adapted to operate under cold conditions. In what follows, we refer to ‘cold imaging’ as optical microscopy performed on live cold-adapted cells at sub-ambient temperatures, ranging between ∼0 °C and 10 °C typical of the physiological environment in which they remain biologically active (Nedwell 1999, Margesin *et al* 2008).

We first examine design considerations for probes optimised for imaging in the cold, followed by a discussion on instrumentation for successful cold microscopy, and finally provide a perspective on research questions that could be unlocked by advancing these systems.

## 2. Fluorophore behaviour and labelling bias at low temperature

Fluorescent probes used for imaging of live biological samples are almost universally optimised and characterised at physiological temperatures at which most human and mammalian cells are cultured (Curd *et al* 2026). For example, commonly used cell lines such as COS-7, HEK, and HeLa cells are routinely maintained and imaged at 37 °C (Ettinger and Wittmann 2014, Kadam *et al* 2017). Nevertheless, many of the key photophysical and biochemical properties of the fluorescent probes are temperature-dependent (Kuzkova *et al* 2014). The practical significance of these temperature-dependent changes will depend on the key observables relevant to the experiment, including spatial resolution, molecular dynamics, diffusion, and kinetics.

Much of our insight into how fluorophores behave as thermal energy is reduced comes from imaging at cryogenic temperatures (Wolff *et al* 2016). At vitrified conditions (∼77 K), reversible photoswitching, photoactivation, and emission spectra are largely preserved across a broad range of fluorescent proteins, with reductions in photobleaching and increases in photon yield per molecule (Tuijtel *et al* 2019). Lowering temperature suppresses non-radiative decay pathways, increasing fluorescence quantum yield and thereby the number of photons emitted per excitation event. Low temperatures also slow diffusive processes and hence the probability that fluorophores encounter reactive oxygen species, thus making irreversible photobleaching less likely. Additionally, lowering temperature reduces intersystem crossing, which reduces blinking and state transitions. An interesting application to benefit from this is structured illumination microscopy (SIM) at cryogenic temperatures, for which increased signal-to-noise ratio and reduced photodamage have been reported (Phillips *et al* 2020, Li *et al* 2023). Cryogenic single-molecule studies furthermore show that photoswitching pathways persist even at extremely low temperatures, but with substantially increased switching timescales (Mazal *et al* 2025).

**Figure 1.**
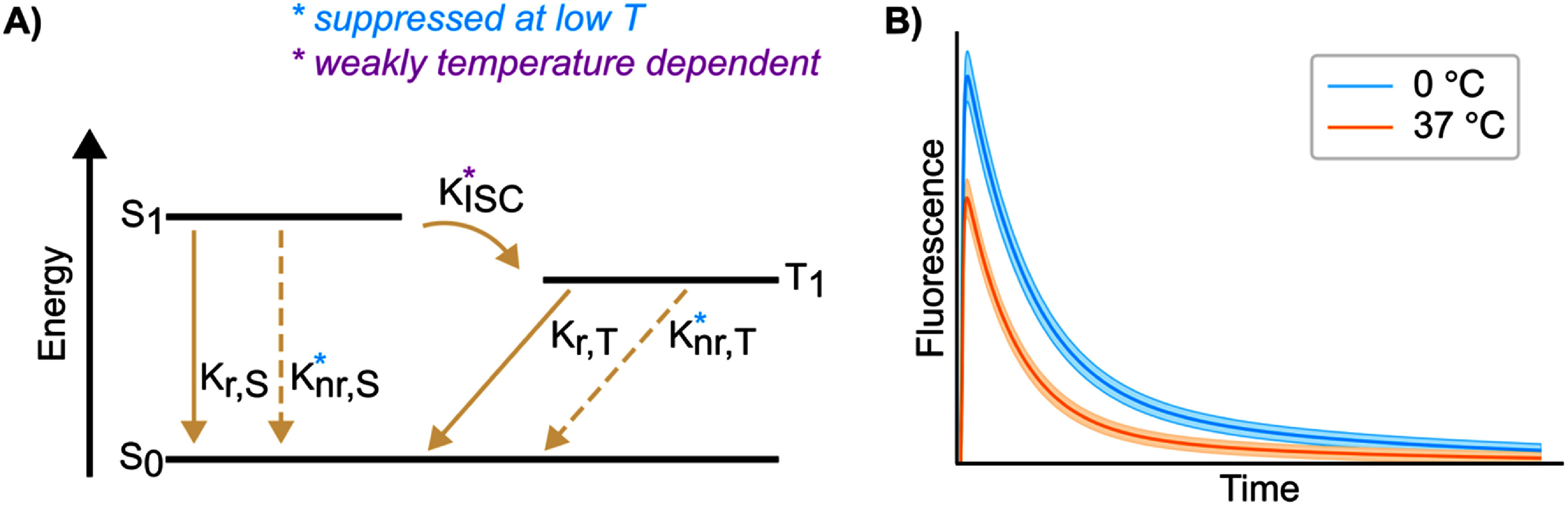
The photophysical properties of the fluorophores are temperature-dependent. (A) Jablonski diagram illustrating radiative and non-radiative transitions relevant to fluorescence emission. At low temperatures, non-radiative relaxation from the singlet excited state (*K*_nr,S_) is suppressed, increasing fluorescence quantum yield. Intersystem crossing (*K*_ISC_) populates the dark triplet states (*T*_1_), from which radiative (*K*_r,T_) and non-radiative (*K*_nr,T_) decay pathways return the molecule to the ground state; *K*_ISC_ is often thermally activated and is therefore reduced at low temperature, decreasing the frequency of dark-state transitions and blinking. The radiative rate from the singlet state (*K*_r,S_) is weakly temperature-dependent. (B) Representative fluorescence intensity decay curves at 0 °C and 37 °C, normalised to the same initial amplitude to isolate differences in decay rate.

Whilst some of these characteristics are retained at 0 °C, they are less pronounced. Properties such as fluorescence quantum yield may still be slightly improved due to suppression of non-radiative decay pathways (figure 1(A)). Many fluorescent proteins were found to retain, and in some cases experience enhancements in, key photophysical properties at low temperature (Mauring *et al* 2005). However, translating such potential advantages to live-cell imaging near 0 °C is non-trivial. Unlike in cryogenic conditions, living cells adapted to the cold retain fluidity, are dynamically heterogeneous, and mechanically active. The viscosity of the cytosolic solvent phase increases with decreasing temperature, and diffusion coefficients decrease accordingly. Moreover, molecular mobility is spatially heterogeneous within cells, as widely reported even in mesophilic systems (Garner *et al* 2023, De Angelis *et al* 2024), but at near-freezing temperatures these intrinsic heterogeneities are likely further amplified, potentially due to reduced thermal energy and increased crowding. As a result, the local environment strongly influences fluorophore behaviour and can modulate, and in some cases outweigh, intrinsic photophysical properties (figure 1(B)). Molecular motion is furthermore reduced, altering cellular properties, such as membrane fluidity (Sezgin *et al* 2017) and cytoplasmic viscosity (Kuimova *et al* 2008, Parker *et al* 2010). These phenomena collectively affect the efficiency of probes to find their target and to engage with them (Lee *et al* 2019), limiting labelling strategies that rely on diffusion-mediated target encounter or lateral mobility within membranes (Kusumi *et al* 2014).

An additional layer of complexity arises from biological cold adaptation. In organisms or cells adapted to low temperatures, target proteins (Berthelot *et al* 2019), membranes (Storelli *et al* 1998), and assemblies may exhibit altered composition or conformational landscapes. Probes validated in mesophilic systems may therefore exhibit weaker target affinity or be affected by different physical or biochemical environments than ones they were optimised for. The behaviour of fluorophores at cold temperatures therefore remains an open field of study, particularly for probes of which the photophysical properties are sensitive to solvent viscosity, refractive index, or molecular crowding. As a result, labelling strategies and image acquisition protocols optimised for 37 °C may not be adequate to report reliably on biological processes at cold.

## 3. Instrumentation constraints for cold imaging

### 3.1. Sample environment engineering

Most temperature-controlled microscopy experiments use a chamber to fully enclose the sample, particularly when cryogenic conditions are required (Hampton *et al* 2017, Karlsson 2021). These chambers offer wide temperature ranges (approximately −50 °C to +100 °C in the most versatile systems) and relatively fast equilibration times (on the order of minutes). However, enclosing the sample increases the working distances for imaging and introduces multiple glass–air interfaces between the objective and specimen. The resulting refractive index mismatches introduce spherical aberrations and loss of high-angle components of the Fourier plane, resulting in lower *x*–*y* resolution, with the PSF increasing up to 1.7 fold axially and 1.2 times in a lateral direction (Diaspro *et al* 2002, Egner and Hell 2006). In the case of optical sectioning, this leads to serious clipping of the fluorescent signal.

These constraints make enclosing the sample in a chamber unsuitable for high- or super-resolution imaging and generally restrict their use to air objectives. Two notable exceptions bypass this limitation but remain fringe examples: (i) a modified water-immersion objective with its front lens mounted in insulating ceramic to reduce heat transfer between the lens and objective body, allowing temperatures as low as −140 °C to be realised (Faoro *et al* 2018); and (ii) a hybrid configuration using two oil-immersion lenses within a cryogenic chamber (−180 °C) (Le Gros *et al* 2009). Both designs used immersion media whose refractive index matched the lens requirements at cryogenic temperatures, namely the liquid refrigerant HFE-7200 and propane respectively. Both designs aimed to maximize resolution and fluorescence signal when imaging vitrified biological samples at cryogenic temperatures. The maximum resolution achieved with chamber-based cooling remains in the range of 500 nm to 1 *μ*m (Buchner *et al* 2007, Seki and Mazur 2008, Pach *et al* 2018, Li *et al* 2020) (figure 3, table 1).

**Table 1.** The most common approaches for cold microscopy come with trade-offs between temperature control and attainable resolution.

	Chamber (Hampton *et al* 2017, Karlsson 2021)	Cooled objective (Marty *et al* 2024, Mehta *et al* 2025)	Cold room (Frolov *et al* 2023)
Thermal stability	±0.01 0 °C	±0.01 °C	Unknown
Thermal range	−150 to +100 °C	−3.5 to +20 °C	N/A
Settling time	80 °C min^−1^	1–0.5 °C min^−1^	<0.1 °C min^−1^ (estimated)
Attainable numerical aperture	0.6–0.9 (estimated)	1.2	0.25–0.40 (estimated)

**Figure 2.**
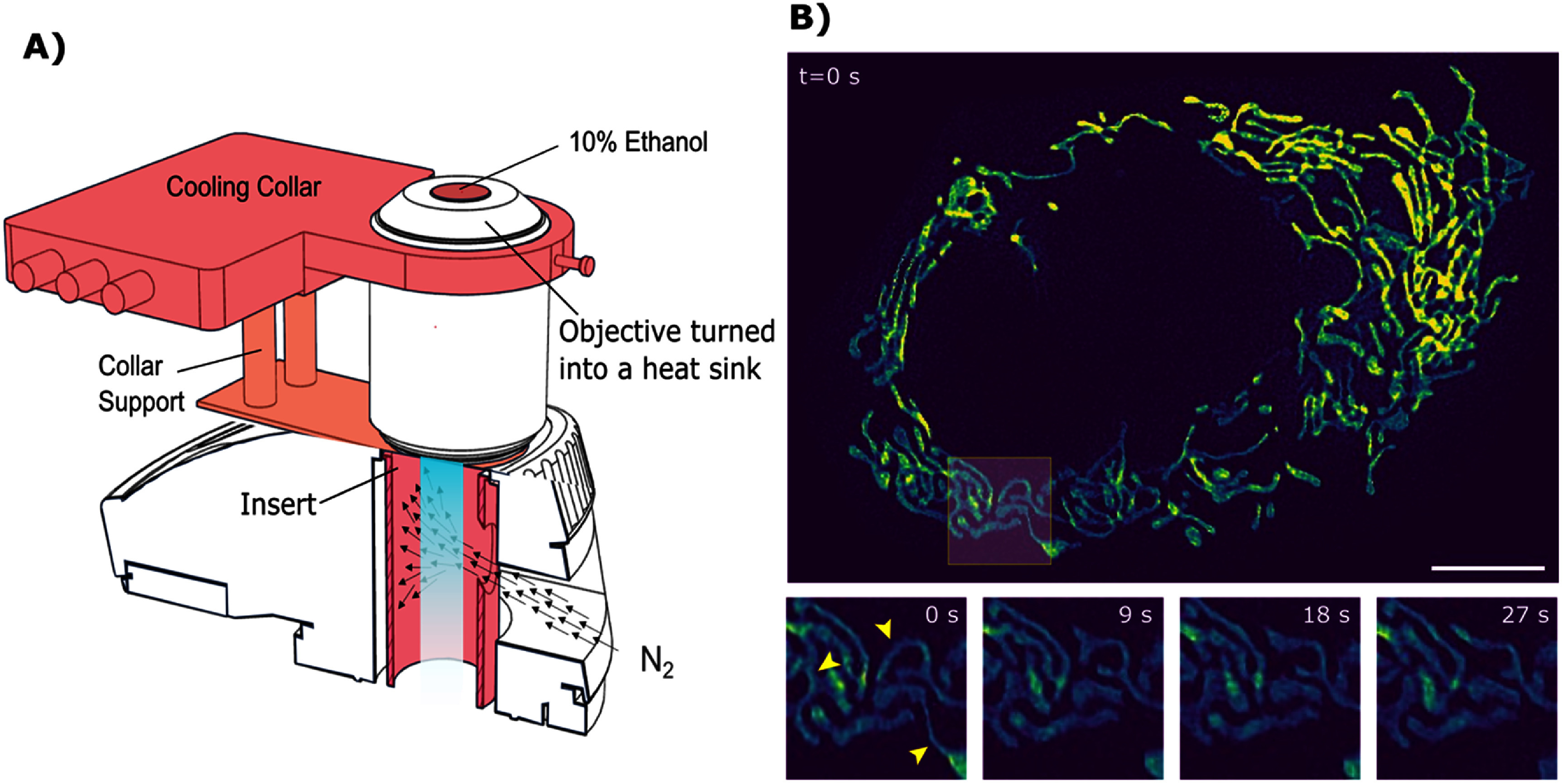
(A) An example of an objective lens cooling collar to enable super-resolution microscopy at low temperature. The design is adapted for use in conjunction with standard microscopy frames and makes them suitable for cold microscopy applications (Reproduced from Marty *et al* 2024. CC BY 4.0). The design enables sample cooling with the objective acting as a heat sink. Nitrogen purging prevents condensation on the back of the lens. (B) Structured illumination microscopy of mitochondria in live, cold-adapted cells, observed at 2 °C using an Olympus 60×/1.2NA water objective. Timelapse images recorded at 9 s intervals illustrate mitochondrial movement in the sample. Yellow arrow tips indicate regions where significant topological changes take place over the time sequence. Scale bar: 5 *μ*m.

**Figure 3.**
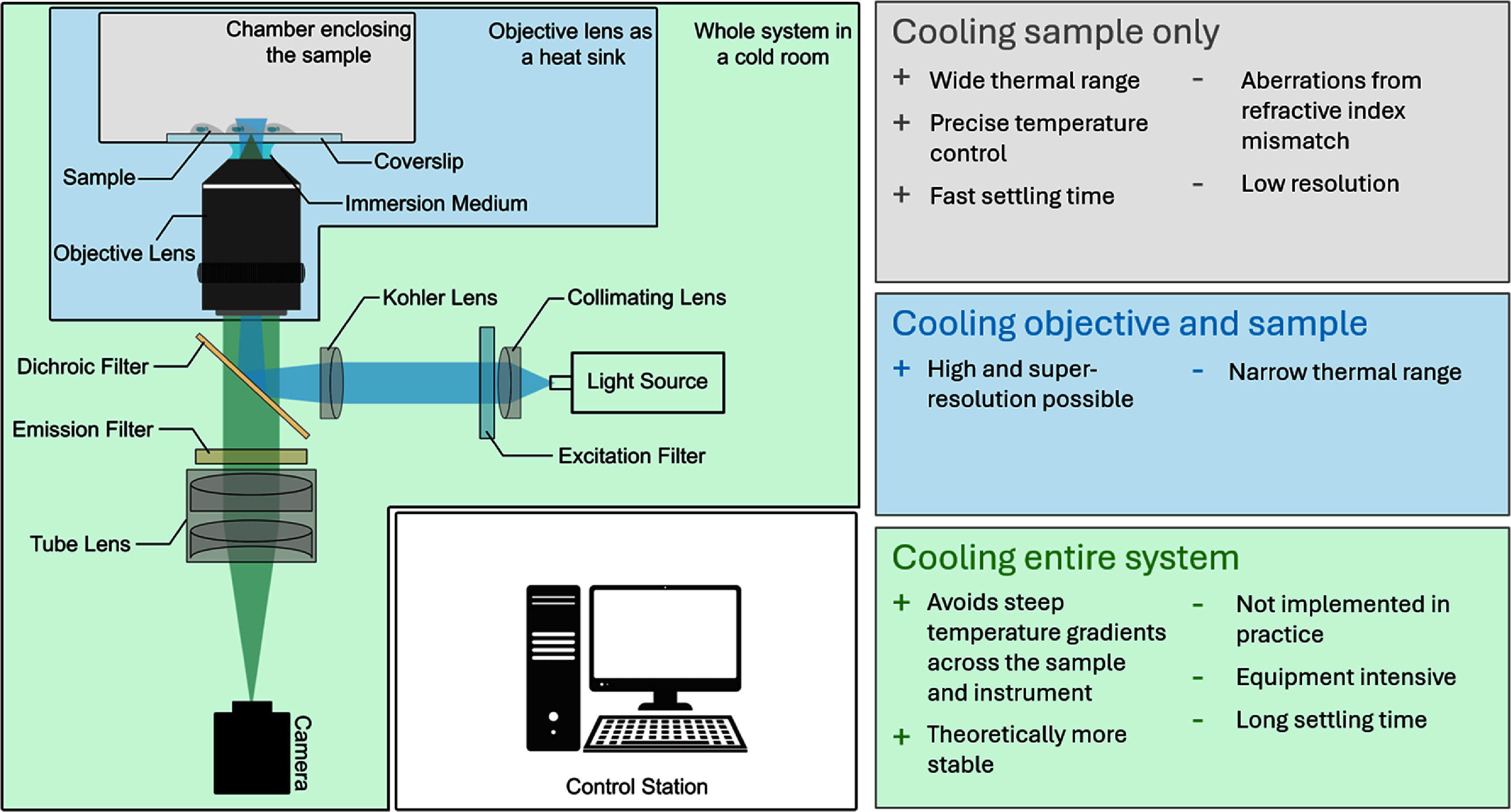
Design options for cold microscopy highlight the trade-off between temperature control and resolution. Cooling only the sample chamber provides the fastest and most precise temperature control, but it is incompatible with high‐resolution imaging. Cooling additional microscope components reduces thermal gradients across the microscope, improving image stability and resolution. However, doing so narrows the usable temperature range for experiments and adds experimental complexity.

High-and super-resolution methods often require contact between the objective lens and the sample through an immersion medium. In these applications, temperature regulation can be achieved by cooling the objective and utilising it as a heat sink, either using in-house (Marty *et al* 2024) or commercial (Mehta *et al* 2025) solutions, and using the immersion medium as a thermal conduit between the sample and the objective lens (figure 2). Cooling the objective can cause condensation at the back of the objective, requiring the use of an objective insulating ring or replacement of humid air by a flow of dry nitrogen. The immersion medium must also be replaced by an appropriate freeze-resistant liquid. Using this method limits the range of achievable temperatures compared to stage- or chamber-based cooling methods, with the lowest reported temperature measured to be −3.5 °C in the sample plane (Marty *et al* 2024). Consequently, lens-cooling strategies are primarily employed to maintain thermal stability during imaging (Rodriguez *et al* 2017, Packer *et al* 2023) rather than to reach temperatures much below −4 °C (figure 3, table 1).

Installing a complete microscopy system in a cold room eliminates issues such as condensation, requirement for use of extended working distances, and refractive index mismatches at glass–air interfaces. However, this method necessitates prolonged thermal equilibration and restricts operational temperature ranges. Furthermore, it requires capability for remote control of the instrumentation, to prevent exposure of the experimenter to cold room conditions. While technically feasible, there is only one documented instance of a complete microscopy setup installed in a cold room (Frolov *et al* 2023) limited to standard widefield light microscopy for the study of ice crystals (figure 3, table 1). The limited adoption of this strategy likely reflects the high cost of dedicating high-end instruments to a single application. A major limitation of building a super-resolution microscope in a cold room is also the complexity of regular system maintenance, for example requirement for optical alignment etc, in a cold environment. Nevertheless, there are some promising opportunities for incorporation of specially designed high-end microscopes into laboratory incubators (Mcclelland *et al* 2025).

### 3.2. Thermal artefacts linked with cooling

• Aberrations from refractive index mismatches

Near-0 °C, the refractive index in aqueous samples changes by ca 5 × 10^−5^ °C^−1^ (Marty *et al* 2024), which can be adjusted for using the objective’s correction collar. The refractive index of ice and glass exhibit smaller thermal sensitivities (approximately 2 × 10^−5^ °C^−1^ and 8 × 10^−6^ °C^−1^ respectively) (Hudgins *et al* 1993, Meyzonnette *et al* 2019). This differential change of refractive index with temperature is not significant across small temperature changes but can produce substantial variations across broader temperature ranges (Hudgins *et al* 1993, Rocha *et al* 2024). Several studies on cold and cryogenic microscopy explore the use of non-standard immersion media to prevent freezing while retaining optical properties (Le Gros *et al* 2009, Faoro *et al* 2018, Marty *et al* 2024). However, no study explicitly addresses the refractive index mismatches arising from the differential thermal dependence of the refractive index across a temperature range.

• Mechanical drift

Under standard laboratory conditions, small variations in room temperature, air currents, and operator proximity have all been known to cause mechanical drift through differential thermal expansion of microscope components. Typical drifts in the range of ∼1 *μ*m (lateral) and ∼3 *μ*m (axial) per 10 °C change have been reported (Adler and Pagakis 2003). Cooling a microscope down from room temperature to near 0 °C thus leads to significant drift. In experiments requiring temperature cycles (e.g. for following freeze–thawing), the problem becomes particularly pertinent. Thermal expansion mismatch between components can introduce defocus and spherical aberrations. The latter may be correctable via the objective collar if within range.

Temperature gradients and the associated drift across the field of view are a known concern for image stability (Adler and Pagakis 2003, Brooks *et al* 2018, Faoro *et al* 2018, Rahmani *et al* 2024). However, systematic measurement of this phenomenon in high-resolution imaging remains rare. Precise quantification of the drift associated with large temperature gradients remains a gap in the literature.

• Stability over many freeze–thaw cycles

Repeated temperature cycling may have long-term effects on equipment that have not been systematically addressed. Since temperature cycling can lead to microstresses and fractures in materials, the long-term stability of components may become compromised. Materials may become brittle and plastics are particularly affected in their properties by temperature (Shashoua 2004, Yvonne 2005). Overall, these effects may degrade microscope performance, etc. Few studies address equipment stability after repeated freeze–thaw cycles on individual instruments (Diller and Cravalho 1971, Reid 1978, Faoro *et al* 2018). However, systematic studies have to our knowledge not been reported.

## 4. Applications enabled by cold imaging across the life sciences

Biology behaves differently in the cold. For some organisms, cold environments are their natural habitat, and cold microscopy allows us to study these species under physiologically relevant conditions. Additionally, cold microscopy serves as a controlled physical perturbation, enabling us to observe what happens when we shift an organism away from its normal physiological temperature. What effects does this have on the inner workings of a cell? Temperature affects a myriad of cellular processes: it changes the energy landscape and kinetics of molecular interactions, affects phase behaviour, rheology, and transport, and can influence the stability of cellular components. At low temperatures, rather than simply slowing the process uniformly, the cell reshapes the energetic landscape of molecular interactions, transport, and phase behaviour. This enables opportunities across multiple application domains, particularly where kinetics and mechanical stability limit the observation.

### 4.1. Cold-adapted samples

One of the most immediate advantages of cold imaging is that it permits the imaging of cells from species that naturally inhabit cold environments under physiologically relevant conditions (Tartwijk *et al* 2026b). Many marine polar species live at temperatures near the freezing point of seawater (approximately −2 to 2 °C) (Peck 2018). Live cells derived from these stenothermal (living in a narrow temperature range) species must be imaged at their physiological temperature to avoid the risks of a temperature-induced stress response or fundamental alterations in cellular organisation and dynamics.

Simple methods to assess cell morphology and health are phase-contrast microscopy for label-free imaging or epi-fluorescence microscopy, which can reveal cells’ morphology, and these have been reported for use on cold-adapted specimens (Sanz *et al* 2015, Zhang *et al* 2025). More modern methods, such as super-resolution microscopy, are much more challenging to perform under cold conditions. SIM, for example, would permit the visualisation of organelle morphology and cytoskeletal organisation in cold samples at two-fold better than diffraction-limited resolution (Gustafsson 2000). At larger spatial scales, tissue- and organism-level imaging provides complementary information that cannot be inferred from isolated cells (although sample preparation protocols become more complex at larger scales). This could be addressed with variants of light-sheet imaging (Fahrbach *et al* 2013, Chang *et al* 2020, Vladimirov *et al* 2021, 2024), for example, to reveal how cellular organisation responds to reductions in temperature.

Particular opportunities exist for the development of single-molecule and single-particle tracking approaches to study cold samples (Kulkarni and Wohland 2026). Processes such as vesicular trafficking, endocytosis, membrane fusion, and organelle contact formation often occur on timescales that challenge the temporal resolution of optical microscopy at 37 °C (Tartwijk *et al* 2026a). However, these processes slow down for cold samples, permitting high fidelity studies of cellular transport at low temperature.

### 4.2. Cold-induced stress and functional perturbations

Imaging at sub-ambient temperatures is not restricted to organisms adapted to cold environments. For cells whose physiological temperatures lie near room temperature or above, cooling can be used as a controlled physical perturbation that alters molecular kinetics, transport, and mechanical properties without immediately compromising cell viability (Betz *et al* 2009, Hanks and Wallace 1949).

Cold exposure can induce stress responses that reorganise cellular architecture. Membrane phase behaviour may shift toward increased order, cytoskeletal dynamics slow down, and protein complexes may become kinetically trapped in non-equilibrium states (Veatch and Keller 2003). These effects are often difficult to resolve at physiological temperature due to rapid relaxation. Imaging under cold conditions can therefore stabilise transient intermediates and reveal spatial heterogeneity and structural states that are otherwise invisible in live-cell experiments at 37 °C.

Importantly, cold-induced perturbations are not purely passive. Cells actively respond to reduced temperature through changes in metabolism, ion homeostasis, stress signalling pathways, and protein synthesis modifications (Lin *et al* 2023). Imaging these responses in real time allows investigation of how cells sense and adapt to altered thermal environments, and how robustness emerges from the coupling between biochemical regulation and physical constraints. Distinguishing between direct physical effects of cooling and active biological adaptation remains a key challenge but also represents a unique opportunity for quantitative imaging.

### 4.3. Astrobiology and extremophile study

A cold imaging microscope that can be integrated with other technological advances such as imaging at microgravity (Wareing *et al* 2025), enables studying biological processes under conditions analogous to extraterrestrial or extreme terrestrial environments. Imaging microbial systems, biofilms, or biomineralisation processes at extreme conditions supports research into habitability, survival strategies, and the limits of life (Cockell *et al* 2020, Castelein *et al* 2021). These applications connect optical microscopy to broader questions in planetary science and astro-microbiology.

## 5. Future directions & unmet needs

Overall, we identify opportunities for methodological development to advance cold optical microscopy. A primary objective is the development of fluorophores specifically optimised for live-cell imaging at low temperatures (0 °C–5 °C). Imaging under these conditions offers the potential for improved signal-to-noise ratios and enhanced photostability. At the same time, it is critical to ensure that the altered physicochemical properties of aqueous samples at low temperature such as increased viscosity and reduced molecular dynamics do not adversely affect probe performance. In addition, fluorophore specificity to their intended targets must remain robust in systems that may differ substantially from canonical 37 °C mammalian models. While a small number of commonly used dyes have demonstrated favourable photostability and signal-to-noise under these conditions, and have been successfully used in cold microscopy studies, this repertoire remains limited. Expanding it will require either systematic testing and reporting of existing fluorophores under cold conditions, or the rational design of new fluorophores with low-temperature constraints explicitly considered.

More versatile instrument designs for cold imaging is also an area of future development. Ideally, hardware for cold optical microscopy should function as modular add-ons that can be integrated into existing microscope platforms without requiring extensive modifications of the optical setup. Current solutions can readily be implemented, but they tend to be optimised either for very low temperatures at comparatively low spatial resolution, or for high-resolution imaging at more moderate temperatures (down to around 0 °C). Further engineering and optimisation are therefore required to bridge this gap and enable routine high-resolution imaging at substantially lower temperatures.

Finally, and perhaps most importantly, cold microscopy opens an avenue for addressing a wide range of fundamental, yet largely unexplored research questions across the biological spectrum. Using temperature as a control parameter, rather than a mere environmental variable, it is possible to study new biophysical mechanisms ranging from transition states of cold-adapted enzymes to complex chemo-physical kinetics that govern life at the cellular scale. Beyond basic science, there are potential applications with transformative potential in medicine, for example for the preservation and storage of tissues and organs, or for the industrial optimisation of biotechnological processes. Lowering the technical barriers to cold microscopy and making these tools more widely available would greatly advance capability in these and other important fields.

## Data Availability

No new data were created or analysed in this study.
